# *Bartonella henselae* infection in a family experiencing neurological and neurocognitive abnormalities after woodlouse hunter spider bites

**DOI:** 10.1186/1756-3305-6-98

**Published:** 2013-04-15

**Authors:** Patricia E Mascarelli, Ricardo G Maggi, Sarah Hopkins, B Robert Mozayeni, Chelsea L Trull, Julie M Bradley, Barbara C Hegarty, Edward B Breitschwerdt

**Affiliations:** 1 Intracellular Pathogens Research Laboratory, Center for Comparative Medicine and Translational Research, College of Veterinary Medicine, North Carolina State University, 1060 William Moore Drive, Raleigh, NC 27607, USA; 2 Department of Neurology, Nemours/AI DuPont Hospital for Children, Wilmington, DE, USA; 3 Translational Medical Group, P.C., North Bethesda, MD, USA

**Keywords:** *Bartonella*, Spiders, Neurological disease, Guillain-Barre Syndrome, Serology, PCR

## Abstract

**Background:**

*Bartonella* species comprise a group of zoonotic pathogens that are usually acquired by vector transmission or by animal bites or scratches.

**Methods:**

PCR targeting the *Bartonella* 16S-23S intergenic spacer (ITS) region was used in conjunction with BAPGM (Bartonella alpha Proteobacteria growth medium) enrichment blood culture to determine the infection status of the family members and to amplify DNA from spiders and woodlice. Antibody titers to *B. vinsonii* subsp. *berkhoffii* (*Bvb*) genotypes I-III, *B. henselae* (*Bh*) and *B. koehlerae* (*Bk*) were determined using an IFA test. Management of the medical problems reported by these patients was provided by their respective physicians.

**Results:**

In this investigation, immediately prior to the onset of symptoms two children in a family experienced puncture-like skin lesions after exposure to and presumptive bites from woodlouse hunter spiders. Shortly thereafter, the mother and both children developed hive-like lesions. Over the ensuing months, the youngest son was diagnosed with Guillain-Barre (GBS) syndrome followed by Chronic Inflammatory Demyelinating Polyradiculoneuropathy (CIDP). The older son developed intermittent disorientation and irritability, and the mother experienced fatigue, headaches, joint pain and memory loss. When tested approximately three years after the woodlouse hunter spider infestation, all three family members were *Bartonella henselae* seroreactive and *B. henselae* DNA was amplified and sequenced from blood, serum or *Bartonella* alpha-proteobacteria (BAPGM) enrichment blood cultures from the mother and oldest son. Also, *B. henselae* DNA was PCR amplified and sequenced from a woodlouse and from woodlouse hunter spiders collected adjacent to the family’s home.

**Conclusions:**

Although it was not possible to determine whether the family’s *B. henselae* infections were acquired by spider bites or whether the spiders and woodlice were merely accidental hosts, physicians should consider the possibility that *B. henselae* represents an antecedent infection for GBS, CIDP, and non-specific neurocognitive abnormalities.

## Background

The genus *Bartonella* is composed of fastidious, Gram-negative and aerobic bacilli belonging to the Alpha proteobacteria group. *Bartonella* species (spp.) are hemotropic, arthropod-borne bacteria that cause long-term bacteremia in mammalian reservoir hosts [1,2]. During the past decade, there has been a dramatic increase in the number of new *Bartonella* species that have been discovered among diverse animal reservoir hosts in geographical regions throughout the world. Since 1990, over thirty *Bartonella* species and sub-species have been characterized and named, with many other putative species yet to be described. Globally, these bacteria reside in diverse ecological niches; many cause persistent intravascular infection in reservoir hosts and 17 *Bartonella* spp. have been associated with an expanding spectrum of human and animal diseases, ranging from acute febrile illnesses to more severe disease manifestations, including encephalopathy, endocarditis, myocarditis, sensory and motor neuropathies, pleural and pericardial effusion, pneumonia, granulomatous hepatitis and hemolytic anemia [3-7].

The natural history for seemingly all *Bartonella* spp. consists of one or more reservoir hosts and one or more transmission competent arthropod vectors. A vertebrate, generally a mammal, sustains a chronic intravascular infection, which in some instances is associated with a relapsing pattern of bacteremia. The persistently infected host serves as the blood reservoir for perpetuation of the transmission cycle, with an arthropod vector transferring the bacteria from the reservoir host to a susceptible uninfected host [8]. Most vectors for *Bartonella* spp. are arthropods. The vector for *B. quintana* is the body louse (*Pediculus humanus* and potentially *Pediculus capitis*) and for *B. bacilliformis* is the sandfly (*Lutzomyia verrucarum*). Fleas (for example *Ctenocephalides felis* on cats and dogs) play a major role in the natural transmission cycle for many *bartonellae*, especially *B. henselae* among pets and wildlife [9,10]. There is also a growing spectrum of arthropods that have been implicated as potential vectors for *Bartonella* species. Genetic diversity and bacterial strain variability appear to enhance the ability of *Bartonella* spp. to infect not only specific reservoir hosts, but also accidental hosts, as has been shown for B*. henselae*[11].

Because neurological abnormalities developed in both children after woodlouse hunter spider bites were suspected by their parents, a family from Kentucky was directed to our laboratory for inclusion in a *Bartonella* research study. Three family members were *B. henselae* seroreactive and *B. henselae* DNA was amplified and sequenced from the mother and older son’s blood, and from a woodlouse and woodlouse hunter spiders.

### Family historical summary

Prior to moving to a new apartment housing location in suburban Louisville, Kentucky on May 1, 2008, all four family members were healthy and had normal sleep patterns. Two months earlier, while in a previous apartment, a bat was removed by an exterminator. Although the bat was flying free within the apartment when the family awakened, there was no indication of bite wounds. The family’s dog had also experienced a flea infestation prior to moving to the new apartment. The family dog was the only pet, there was no history of family members experiencing bites or scratches and no flea infestations were reported after moving into the new apartment or subsequently to a new house in the same neighborhood.

In July 2008, their new apartment flooded, after which there was a large influx of woodlice (order Isopoda). Subsequently, the mother reported seeing occasional wood louse hunter spiders (*Dysdera crocata*) in the apartment, including in the children’s beds and on the children. During August 2008, the parents suspected that both sons (5 months-old and 5 years-old, respectively) were bitten by woodlice hunter spiders. The mother (41 years-old) did not knowingly experience any spider bites. After the apartment was treated by an exterminator, no woodlice or woodlice hunter spiders were observed in or around the apartment. Subsequently, the mother and both sons developed recurrent rash-like skin lesions, disruptive sleep patterns and both boys developed anxiety accompanied by episodes of inconsolable crying, irritability, and panic attacks. In July 2009, the oldest son was examined by a surgeon because of enlarged lymph nodes in the neck. Over the ensuing months, the mother developed symptoms, including fatigue, headaches, joint pain, eye pain, insomnia, memory loss, disorientation, irritability, weakness in the upper extremities and loss of sensation to both legs. In May 2010, as detailed in the case report below, the youngest son was diagnosed with Guillain-Barre syndrome (GBS), and subsequently with Chronic Inflammatory Demyelinating Polyneuropathy (CIDP).

After contacting the corresponding author and describing the family's medical history, the mother elected to enter her sons and herself into an ongoing study regarding *Bartonella* spp. infection in patients with arthropod and animal exposures. (North Carolina State University Institutional Review Board approval IRB 1960-11). Beginning in August 2011, blood and serum samples from the mother, both sons and the dog were submitted for *Bartonella* testing. As the parent’s initial concerns related to the youngest son’s CIDP diagnosis, this child was tested in August 2011, followed by the mother and dog in November, and the oldest son in April 2012. The father did not recall being bitten by a spider himself, remained healthy during the course of this investigation and was never tested for evidence of *Bartonella* sp. infection. Over a one-year period (2011–2012), spiders, identified as wood louse hunter spiders (*Dysdera crocata*), and several woodlice (Isopoda order) collected from around the family’s new house (located three miles from the spider infested apartment) were submitted by express mail for manual DNA extraction and *Bartonella* PCR.

## Methods

### Samples

Aseptically obtained EDTA (ethylene diaminetetraacetic acid)-anti-coagulated blood and serum samples from the family, their dog, whole spiders and woodlice collected from around the apartment in Kentucky, were submitted to the North Carolina State University College of Veterinary Medicine Intracellular Pathogens Research Laboratory (NCSU-CVM-IPRL) for *Bartonella* testing. Collection and analyses of these data were conducted in conjunction with North Carolina State University Institutional Review Board approval (IRB no. 1960-11).

### Serological analyses

*Bartonella vinsonii* subsp. *berkhoffii* genotypes I, II, III, *B. henselae* (Houston 1strain), *B. henselae* (San Antonio 2 strain), and *Bartonella koehlerae* antibodies were determined in the Intracellular Pathogens Research Laboratory (IPRL) following traditional immunofluorescence antibody assay (IFA) practices with fluorescein conjugated goat anti-human IgG (Pierce Biotechnology Rockford IL), as described in previous studies from our laboratory [12-14]. *Bartonella* organisms of feline isolates of *B.koehlerae* (NCSU 09FO-01) and *B.henselae* H-1 (NCSU 93FO-23), *B. henselae* SA2 (NCSU 95FO-099) and canine isolates of *B.vinsonii berkhoffii* genotype I (NCSU 93CO-01), II (NCSU 95CO-08) and III (NCSU 06CO-01) were passed from agar grown cultures into cell cultures to obtain antigens for IFA testing Heavily infected cell cultures were spotted onto 30-well Teflon coated slides (Cel-Line/Thermo Scientific), air dried, acetone fixed and stored frozen. Serum samples were diluted in phosphate buffered saline (PBS) solution containing normal goat serum, Tween-20 and powdered nonfat dry milk to block non-specific antigen binding sites. Sera were screened at dilutions of 1:16 to 1:8192. To avoid confusion with possible non-specific binding found at low dilutions and to standardize with other laboratories such as the CDC, a cutoff titer of 1:64 was used to define a seroreactive titer.

### DNA extraction, PCR assay and DNA sequencing

A previously described approach that combines PCR amplification of *Bartonella* spp. DNA from blood, serum and enrichment BAPGM (*Bartonella* alpha Proteobacteria growth medium) enrichment blood culture was used to test EDTA-anti-coagulated whole blood and centrifuged serum samples [4,13,15-17]. DNA was automatically extracted from 200 ul of EDTA-anticoagulated blood, from serum and from 200 ul of BAPGM enrichment blood culture, using a BioRobot Symphony Workstation and MagAttract DNA blood kit (Qiagen, Valencia, CA). Prior to extraction of DNA from the woodlice and wood louse hunter spiders, each individual specimen was washed twice using 2 ml of dH_2_O followed by a single wash with 95% ethanol. For DNA extraction, the entire body of each arthropod was pulverized to a fine powder by bead-beating using stainless steel beads. DNA from spiders and woodlice were manually extracted using DNeasy blood and tissue mini kit following manufacturer’s instructions (Qiagen, Valencia, CA). *Bartonella* DNA was amplified using conventional *Bartonella* genus PCR primers targeting the 16S-23S intergenic spacer region (ITS) as previously described [18,19]. *Bartonella* genus PCR was performed using oligonucleotides 425 s (5^′^CCGGGGAAGGTTTTCCGGTTTATCC 3^′^), 325 s (5^′^ CCTCAGATGATGATCCCAAGCCTTTTGGCG 3^′^) and 1000as (5^′^ CTGAGCTACGGCCCCTAAATCAGG 3^′^) as forward and reverse primers, respectively. Amplification was performed in a 25-ul final volume reaction containing 12.5 uL of MyTaq Premix (Bioline), 0.2 uL of 100 umol/L of each forward and reverse primer (IDT^®^DNA Technology, Coralville, IA, USA), 7.3 uL of molecular grade water, and 5 uL of DNA from each sample tested. Conventional PCR was performed in an Eppendorf Mastercycler EPgradient^®^ (Eppendorf, Hauppauge, NY, USA) under the following conditions: a single cycle at 95°C for 2 s, followed by 55 cycles with DNA denaturing at 94°C for 15 s, annealing at 66°C for 15 s, and extension at 72°C for 18 s. The PCR reaction was completed by a final cycle at 72°C for 30s. All PCR reactions were analyzed by 2% agarose gel electrophoresis. Amplicons obtained from arthropod and human samples were sequenced to identify the *Bartonella* sp. and ITS strain type. Bacterial species and strains were defined by comparing similarities with other sequences deposited in the GenBank database using the Basic Local Alignment Search Tool (Blast version 2.0).

## Results

### Individual medical histories

#### Youngest son

**Figure 1:**
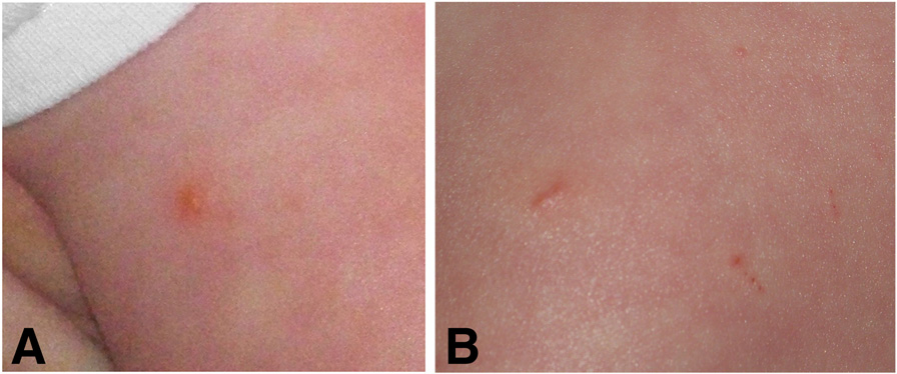
**Photographs taken by the parents during the woodlouse and woodlouse hunter spider infestations. **Puncture-like bite lesions were observed in the skin overlying the mid humerus (**A**) and proximal femur (**B**).

In August 2008, the youngest son, a developmentally normal child with precocious motor skills, sustained puncture-like bite lesions in the skin overlying the mid humerus and proximal femur (Figure 1). Based upon the exposure history and appearance of the lesions, spider bites were diagnosed by the child’s pediatrician. Subsequently, the boy developed intermittent rashes that were initially diagnosed as food allergy and was concurrently diagnosed with chronic sinusitis. At approximately 24 months of age, (February 2010), his parents first noted that he would stumble. He also awakened at night crying and complaining of pain in his legs. Other concerns included early morning awakenings, constipation, intermittent complaints of dizziness, and seeing “spots.”

In May 2010, about a month after an upper respiratory infection, the boy was unable to climb stairs. His parents brought him to an emergency room, where it was noted that he was unable to stand from a seated position on the floor. A lumbar puncture revealed an elevated CSF protein of 110 (normal 15–45) with 4 white blood cells. An MRI of the spine demonstrated enhancement of the ventral nerve roots and pial enhancement from the 11^th^ thoracic vertebrae through the remainder of the spinal cord. Neurological examination was significant for areflexia and weakness in the lower extremities. Guillain-Barre Syndrome was diagnosed and he was treated with 2 grams per kilogram of intravenous gammaglobulin (IVIG) over 4 days. He improved rapidly and was discharged following the infusions.

When re-examined one month later, his leg strength was judged to be improved to 85% of normal and no further treatment was given. By July 2010, he was less able to walk without stumbling and was unable to stand from a seated position. He also complained of tingling and discomfort around his mouth and pain in the legs. IVIG was administered at a dose of 1 gram/kilogram on each of 2 days. Electromyelogram (EMG) findings were consistent with chronic sensory motor demyelinating polyneuropathy with secondary axonal features and conduction block. CIDP was diagnosed and IVIG treatments were re-instituted every 4 weeks, along with a 4 week course of prednisone and gabapentin for pain. By May 2011, following internet searches, his mother became concerned that the boy’s symptoms might be related to *Bartonella* infection. Due to this concern, treatment with azithromycin was initiated for 10 days, and it was felt that this was associated with some improvement. *Bartonella henselae* IgM and IgG antibodies were not detectable at a 1:16 dilution (ARUP Laboratories, Salt Lake City Utah). By July 2011, despite medical therapy, pain and the right foot drop had worsened.

When he had another relapse of muscle weakness, IVIG was administered on August 19, 2011, ten days prior to obtaining blood for additional *Bartonella* testing at the NCSU-CVM-IPRL. While awaiting test results, treatment with azithromycin for 30 days was reinitiated. The family felt that they noted almost immediate improvement of the patient’s symptoms and requested that the next IVIG treatment be delayed. The boy was seroreactive to multiple *Bartonella* spp. antigens (see below, Table 1). A decision was made to decrease the dose of the IVIG to 1 g/kg and stretch the infusions to every 6 weeks with the first infusion at this dose being administered in October 2011. It was rapidly clear that this would not be successful as weakness and symptoms of burning pain in his legs returned. Infusions were then reinitiated at a dose of 2 g/kg every 4 weeks and then stretched to every 5 weeks in July 2012. The addition of prednisone was avoided due to concern that immune suppression might interfere with therapeutic elimination of the suspected *Bartonella* infection. After IVIG infusion was reinitiated at the full dose in late 2011 the patient’s muscle strength continued to improve. In May 2012, treatment with clarithromycin 125 mg twice daily and rifampin 150 mg twice daily was instituted. By July 2012, his deep tendon reflexes had returned and his strength was normal. As his pain was diminished, gabapentin was discontinued. Clarithromycin and rifampin have been well tolerated and both drugs were continued through November 2012. As of this writing, the child is ambulating normally, but still occasionally complains of stiffness and joint pain in his legs, particularly when he wakes up in the morning. As of January 31, 2013, polyneuropathy remains in remission and IVIG has not been administered since September 14, 2012. The parents report that the child is actively socializing with other children and now runs and plays like he had never done before.

**Table 1:** Serological, PCR and culture results from the three patients and their family dog

**Family members**	**Date**	**Sample**	**PCR/BAPGM culture results**^**a**^		***Bartonella *IFA reciprocal titers**					
			**Direct**	**Enrichment**	***Bvb *I**	***Bvb *II**	***Bvb *III**	***Bh*HI**	***Bh*SA2**	***Bk***
			**extraction**	**culture**						
Youngest Son	8/29/2011	Serum	Neg	N/D	<16	<16	<16	256	256	128
		Blood	Neg	Neg						
	8/31/2011	Serum	Neg	N/D	64	1024	128	512	512	2048
		Blood	Neg	Neg						
	9/2/2011	Serum	Neg	N/D	32	512	128	256	256	512
		Blood	Neg	Neg						
	11/21/2012	Serum	Neg	Neg	<16	<16	<16	<16	64	<16
		Blood	Neg	Neg						
Mother	11/14/2011	Serum	Neg	N/D	<16	32	<16	<16	64	<16
		Blood	Neg	Neg						
	11/16/2011	Serum	*Bh*	N/D	32	128	64	64	64	32
		Blood	Neg	Neg						
	11/18/2011	Serum	*Bh*SA2	N/D	32	256	32	32	64	64
		Blood	Neg	Neg						
Oldest Son	4/16/2012	Serum	Neg	N/D	<16	256	<16	<16	128	<16
		Blood	Neg	Neg						
	4/18/2012	Serum	Neg	N/D	<16	256	<16	<16	128	<16
		Blood	Neg	*Bh*SA2						
	4/20/2012	Serum	Neg	N/A	<16	256	<16	<16	128	<16
		Blood	*Bh*SA2	Neg						
Dog	11/23/2011	Serum	N/D	N/D	<16	<16	<16	<16	<16	<16
		Blood	Neg	Neg						

^a ^Denotes 16S-23S ITS DNA sequence results.

*Bh = Bartonella henselae, Bh *SA2 *= Bh* strain San Antonio, *Bh *HI *= Bh *strain Houston I.

*Bvb *I, II, and III = *Bartonella vinsonii *subsp. *berkhoffii *genotype I, II, and III.

*Bk* = *Bartonella koehlerae.*

N/D = Not done.

#### Mother

The mother, who takes care of her children and home full-time, was healthy prior to August 2008. She reported limited exposure to cats, wildlife or production animals, but did allow the family dog to sleep in her bed. Prior vector exposure was infrequent, but included fleas, ticks and mosquitoes. She did not recall being bitten by a spider. Subsequent to the spider infestation of the apartment, she developed fatigue, memory difficulties, headaches, irritability, eye pain, insomnia, chest pain, blurred vision, shortness of breath, rash and skin lesions and anxiety attacks. She had also experienced a loss of sensation in her legs, joint pain involving the shoulders and ankles, ear pain, and she frequently had a sore throat. In July 2009, she was examined due to an abdominal rash and shingles was tentatively diagnosed. The mother reported that her symptoms persisted between 2008–2011, without notable improvement or deterioration, during which time she sought care from her family physician, an otolaryngologist and a neurologist. Using blood and serum samples submitted in November 2011, infection with *B. henselae* (SA2 strain) was confirmed serologically and by PCR amplification and DNA sequencing. Between February and July 2012, she was treated with doxycycline 200 mg once daily and rifampin 300 mg twice a day. Following this antibiotic course, the mother reported substantial overall improvement and was almost symptom free. However, she continues to experience occasional irritability, confusion, dizziness, nausea, and pain involving the shoulder, hip and the bottoms of her feet.

#### Oldest son

In August 2008, the older son, was examined by his pediatrician due to suspected spider bites and a rash. This child also had occasional exposure to cats and dogs and had exposure to fleas and mosquitoes. Subsequently, the boy complained of a sore throat, occasional ear pain and pain in the thigh region. In July 2009, the boy’s parents sought medical consultation with a dentist and surgeon for a swollen lymph node in the neck that had persisted for approximately 3 months. The lymph node regressed in size without therapy. During 2009–2012, the parents indicated that the boy experienced episodes of unexplained depression, irritability, and anxiety, but was otherwise healthy. Infection with *B. henselae* SA2 strain was confirmed by serology and BAPGM enrichment blood culture PCR in April 2012. Treatment with clarithromycin 250 mg twice daily was instituted in May 2012. On August 29, 2012, rifampin 300 mg twice daily was added to the treatment regimen, which has continued through November, without known adverse side effects. The sore throat, ear and eye pain resolved by October; however, during the fall 2012 school year, his parents reported increased irritability and rage episodes. In addition, the boy’s teacher indicated a lack of attention during class, and suggested that the child might have an Attention Deficit Hyperactivity Disorder (ADHD). After consultation with the attending physician and a psychiatrist, the parents declined therapy for ADHD.

#### Bartonella spp. serology and BAPGM enrichment PCR results

*Bartonella* serology and PCR results for the three family members and the dog are summarized in Table 1. The youngest son was seroreactive to *B. henselae* SA2, *B. henselae* HI and between August 29^th^ and August 31^st^, there was a four-fold or greater increase in antibody titers to *B. koehlerae* and to *B. vinsonii* subsp. *berkhoffii* genotypes I, II, and III. *Bartonella* sp. DNA was not amplified from his blood, serum or BAPGM enrichment blood cultures. The mother was seroreactive to *B. henselae* HI, *B. henselae* SA2, *B. vinsonii* subsp. *berkhoffii* genotypes II and III and *B. koehlerae*. *Bartonella henselae* bacteremia was confirmed in the mother by PCR amplification from two BAPGM enrichment blood cultures. Based upon the amplified DNA sequences, the *B. henselae* strain in the mother’s samples were 99.8% and 100% similar, respectively *to B. henselae* SA2, (GenBank accession AF369529). The oldest son was seroreactive to *B. henselae* SA2 and *B. vinsonii* subsp. *berkhoffii* genotype II. Based upon the DNA sequences amplified from a blood sample and BAPGM enrichment blood culture sample, the oldest son was bacteremic with a *B. henselae* SA2 strain (99.2% and 99.6% homology with GenBank accession AF369529). The dog was seronegative to all *Bartonella* spp. antigens and no *Bartonella* DNA was amplified from blood or the BAPGM enrichment blood culture. Following BAPGM enrichment culture, no subculture isolates were obtained from any family member. When retested in November 2012, the youngest son was only seroreactive to the *B. henselae* SA2 strain and *Bartonella* sp. DNA was not amplified from blood, serum or the BAPGM enrichment blood culture.

#### PCR testing of woodlice and wood louse hunter spiders

*Bartonella henselae* SA2 DNA (97.0% homology with GenBank accession AF369529) was amplified and sequenced from pooled woodlice (Table 2). Of the thirteen wood louse spiders tested, *B. henselae* SA2 DNA (100% and 99.3% homology, Gen Bank accession AF369529) was amplified and sequenced from two spiders and *B. vinsonii* subsp. *berkhoffii* genotype III DNA (98.6% homology, GenBank accession DQ059765) from one spider. As the family had moved from the apartment in which the bites occurred into a house, all of the spiders and woodlice were collected approximately 3 miles from the original suspected spider bite location.

**Table 2:** ***Bartonella *PCR from spiders and pooled woodlice**

**Spider #**	**Collection date**	**PCR results**^**a**^	**Woodlouse #**	**Collection date**	**PCR results**^**a**^
1	9/28/2011	*Bh* SA2	1	9/8/2011	Neg
2		Neg	2		Neg
3		*Bvb* III	3		Neg
4		Neg	4		*Bh* SA2
5		Neg			
6	6/1/2012	Neg			
7		Neg			
8		Neg			
9	9/12/2012	Neg			
10		Neg			
11	10/8/2012	*Bh* SA2			
12		Neg			
13		Neg			

^a ^Denotes 16S-23S ITS DNA sequencing results.

*Bh *SA2 = *Bartonella henselae *strain San Antonio 2.

*Bvb *III = *Bartonella vinsonii *subsp. *berkhoffii *genotype III.

## Discussion

PCR amplification and sequencing of *B. henselae* SA2 DNA from two family members, woodlouse hunter spiders, and a woodlouse collected at least three years after family members were exposed to and the children were presumably bitten by similar spiders, was unexpected. To the best of our knowledge, this is the first report of the presence of *Bartonella* spp. DNA in spiders or in woodlice. Although *B. henselae* DNA was amplified from two spiders collected 13 months apart, a woodlouse, and from serum, blood and BAPGM enrichment culture samples from two family members, these results should be interpreted with caution, as it is not clear whether *Bartonella* was acquired at the time of the infestation and spider bites or whether the spiders and woodlice are accidental hosts for *Bartonella* spp. Since the woodlouse hunter spider is thought to feed exclusively on woodlice (a land-dwelling crustacean), the amplification of *Bartonella* DNA from spiders and woodlice suggests that the *B. henselae*-infected spiders fed on infected woodlice. Preliminary results (unpublished data) obtained in our laboratory indicates that washed woodlice can become PCR-positive for *B. henselae* after feeding on food contaminated with the bacteria. Although the length of time that *B. henselae* can remain viable within the environment has not been investigated to any degree, the bacteria remains viable in flea feces for several days. Whether bacteria ingested by woodlice remain viable, whether replication can occur, how long a *Bartonella* sp. can be retained within the isopod and whether a spider feeding on this crustacean can acquire or transmit *Bartonella* are subjects for future studies. Although the family experienced a flea infestation prior to moving into the new apartment, the family dog was not seroreactive to *Bartonella* sp. antigens and was PCR negative in blood and BAPGM enrichment blood culture, making the dog and potentially fleas a less likely source of *B. henselae* transmission to family members. To date, *B. henselae* has not been reported in bats to the author’s knowledge, no family member experienced a bat bite, and the bat exposure occurred several months before the onset of illness in the children and mother.

*Bartonella* DNA has also been amplified from non-hematophagous arthropods, such as honey bees [20]. Those authors hypothesized that honey bees ingested or acquired *Bartonella* organisms through environmental contact. In a recent report, a patient with neuroretinitis, a well documented ocular pathology induced by *B. henselae*, was diagnosed with bartonellosis following the bite (sting) of a bull ant (genus *Myrmecia*) in Australia [21]. These authors suggested that *B. henselae* was probably transmitted to the patient via the stinger or mandibles, which provided a portal for bacterial entry into the skin. These recent publications indicate that physicians should routinely review a patient’s medical history for arthropod exposure. Based upon recent clinical and research observations, there appears to be a growing spectrum of arthropods that might serve as vectors for *Bartonella* species, thereby emphasizing the critical importance of and the need for additional experimentally controlled vector competence studies. In addition, localizaton of *Bartonella* sp. replication within arthropods, further documentation of other potential animal reservoirs, and the determination of trans-ovarian transmission in various arthropod species represent other important issues that require scientific attention.

From a clinical perspective, the non-specific symptoms reported in the mother are consistent with previous reports of *Bartonella* sp. bacteremia in immunocompetent patients [4,22]. Although less well characterized, the behavioral and neurocognitive abnormalities that predominated in the older son have also been reported in *Bartonella* bacteremic children [14,23,24]. Interestingly, and as reported in a small subset of patients in two case series, *B. henselae* DNA was only amplified from the mother's extracted serum samples, whereas *B. henselae* DNA was amplified from both blood and a BAPGM enrichment blood culture from the oldest son [4,22]. The reason(s) for these observations remain unclear, but one study has reported progressive increases in serum DNA concentration in association with prolonged sample storage times in certain pathologic conditions [25]. *Bartonella* DNA was never amplified from a negative control and DNA from a *B. henselae* H1 strain (not *B. henselae* SA2 as found in this study) was used as a positive control for all PCR testing, therefore laboratory contamination is an unlikely explanation for the PCR and DNA sequencing results reported in this study. Due to the fact that *B. henselae* induces a relapsing bacteremia in cats [26] and *B. birtlesii* induces a relapsing bacteremia in experimentally infected rodents [27], three blood samples obtained at approximately 2 day intervals were tested for each patient. For the mother and oldest son, only two dates yielded positive PCR results, potentially supporting the possibility of a relapsing pattern of *B. henselae* bacteremia in human patients. Also, as reported previously from our laboratory [28], there was considerable variability in the mother’s and youngest son’s antibody titers when serum samples obtained within a one-week time frame were tested using an IFA technique. The mother had low antibody titers with up to four-fold variations in four of the six *Bartonella* spp. antigens over a one week period. The youngest son had identical antibody titers to *B. henselae* strains H1 and SA2, but seemingly seroconverted to *B. vinsonii* subsp. *berkhoffii* genotypes I, II III and *B. koehlerae*. Administration of IVIG ten days prior to collection of the initial blood sample may well have influenced the youngest son’s serological results, particularly if IVIG has antibacterial properties [29]. In contrast, the oldest son’s antibody titers were identical for all six antigens at all three time points. In the context of antigenic specificity, he had antibodies to *B. henselae* SA2 strain, but not to a *B.henselae* H1 strain. All serum sample sets from each patient were tested at the same time, by the same experienced technician, using the same conjugate and IFA antigen slides. Whether these serological discrepancies are related to sample collection and storage issues, a prozone effect associated with excess antigen, IVIG or other unknown factors requires additional investigation.

Similar to the initial diagnosis in the youngest son, GBS due to neurobartonellosis was diagnosed in a 10-year-old girl, who was hospitalized due to progressive leg weakness [30]. Seven days earlier, the girl had a self-limiting episode of fever and vomiting of 1 day duration. Four days later, she had difficulty walking, became irritable and complained of severe myalgia in the lower limbs. Laboratory findings were not remarkable. Nerve conduction studies identified decreases in motor conduction velocity and amplitude, consistent with axonal damage. An exhaustive search for known causes of GBS was negative. The girl was treated with IVIG for 5 days, and within two weeks her neurological status had normalized. There was no history of cat scratches, no palpable lymphadenopathy and no hepatic or splenic lesions on an abdominal ultrasound, however, because she lived in a rural area and played with kittens, *B. henselae* serology was requested. Her *B. henselae* IgG titer was 1:1024 and a specific IgM titer was “positive”, although a value was not reported. Her convalescent IgM titer was negative and the IgG antibody titer had decreased. To date, CIDP has not been associated with *Bartonella* infection. Although serology supported *Bartonella* exposure in the younger son, prior administration of IVIG complicates interpretation of his antibody titers and potentially his BAPGM enrichment culture PCR test results. It is possible that the source of *Bartonella* antibodies was the IVIG and that repeated immunoglobulin administration suppressed the level of bacteraemia below the level of successful PCR amplification. CIPD, also referred to as relapsing polyneuropathy, is a neurological disorder characterized by progressive weakness and impaired sensory function in the legs and arms. As was true in the boy in this report, CIPD is often diagnosed as the chronic counterpart of GBS. Prior infection or vaccination can precipitate GBS, and *Campylobacter jejuni* has become the most well recognized antecedent infection [31]. Consideration should be given to *B. henselae* as an antecedent infection for GBS and CIPD. Physicians should pursue the medical history in these patients to determine if they have experienced animal bites or scratches or arthropod bites or stings.

As scientists, physicians and veterinarians learn more about the medical importance of the genus *Bartonella*, there has been enhanced focus on known and suspected arthropod vectors. Because of their ability to reside within erythrocytes of a diverse number of mammalian hosts in conjunction with their diverse ecological niches, there is the potential opportunity for various *Bartonella* spp. to be transmitted by a variety of arthropod vectors. Several blood-feeding arthropods, *Lutzomyia verrucarum*, *Pediculus humanus humanus*, *Ctenocephalides felis* and some rodent fleas (*Ctenophthalmus nobilis*) have been confirmed to be competent vectors for transmission of *Bartonella* species [32]. Tick transmission of *Bartonella* spp. has been a controversial subject in recent years [33,34]; however, vector competence for tick (*Ixodes ricinus*) transmission of a *Bartonella* sp. was recently demonstrated experimentally, thus supporting the possibility that *Ixodes* sp. ticks are transmitting *Bartonella* spp. throughout the northern hemisphere [35]. Previous studies from Europe and North America have documented the presence of *B. henselae* DNA in *Ixodes ricinus*[36]*Ixodes scapularis*[37] and *Ixodes pacificus*[38]. In conclusion, it must be stressed that there is an important difference between the vector competence and vector potential of arthropods from which *Bartonella* spp. DNA is amplified. The amplification of *Bartonella* spp. DNA in the woodlouse hunter spiders in this study does not provide definitive proof of vector competence and may merely represent an accidental infection associated with ingestion of *Bartonella*-infected blood from an infected host (isopod). Although *B. henselae* was amplified and sequenced from woodlouse hunter spiders and from their associated prey, the woodlouse, definitively establishing the source of bacterial transmission to this family was not possible.

## Conclusions

There appears to be a growing spectrum of arthropods that might serve as vectors for various *Bartonella* species. The location of *Bartonella* replication within arthropods, the documentation of other potential reservoirs, and the determination of trans-ovarian transmission in various arthropod species represent important public health issues that need to be resolved. As *B. henselae* SA2 DNA was amplified from woodlouse spiders and from a woodlouse collected nearly three years after the reported bites, it is not clear if the *B. henselae* infections in this family were acquired by spider bites or whether spiders and woodlice were accidental hosts. Also, additional studies are needed to determine whether *B. henselae* bactermia can predispose patients to GBS, CIDP and neurocognitive abnormalities.

### Consent

Written informed consent was obtained from the patient for publication of this report and any accompanying images. The parents contacted the investigators to be entered into an ongoing IRB approved research study and were full supportive of investigations described in this manuscript.

## Abbreviations

GBS: Guillain-Barre syndrome; CIDP: Chronic inflammatory demyelinating polyneuropathy; BAPGM: Bartonella alpha proteobacteria growth medium; EDTA: Ethylene diaminetetraacetic acid; IPRL: Intracellular pathogens research laboratory; EMG: Electromyelogram; IVIG: Intravenous gammaglobulin; ADHD: Attention deficit hyperactivity disorder; IFA: Immunofluorescence antibody assay.

## Competing interests

In conjunction with Dr. Sushama Sontakke and North Carolina State University, Dr. Breitschwerdt holds U.S. Patent No. 7,115,385; Media and Methods for cultivation of microorganisms, which was issued October 3, 2006. He is the chief scientific officer for Galaxy Diagnostics, a company that provides diagnostic testing for the detection of *Bartonella* species infection in animals and human patients. Dr. Ricardo Maggi has lead research efforts to optimize the BAPGM platform and is the Scientific Technical Advisor for Galaxy Diagnostics. Dr. Robert Mozayeni was one of the attending physicians for the patients described in this study and has recently joined Galaxy Diagnostics as the chief medical officer. All other authors have no potential conflicts.

## Authors’ contributions

PM and RM performed the BAPGM enrichment blood culture and PCR testing of the patient samples, woodlice and spiders, performed DNA sequencing and alignments, and generated the first draft of the manuscript. SH is a pediatric neurologist who cared for the youngest son and drafted his case report. BRM is a rheumatologist who cared for the mother and oldest son and drafted their case reports. CT performed PCR testing of the woodlice and spiders. JB and BH assisted in sample acquisition and serological testing. EB coordinated various aspects of the investigation and helped to draft the final manuscript. All authors read and approved the manuscript.

## References

[B1] Chomel BB Boulouis HJ Breitschwerdt EB Kasten RW Vayssier-Taussat M Birtles RJ Koehler JE Dehio C Ecological fitness and strategies of adaptation of Bartonella species to their hosts and vectors Vet Res 2009 40 29 10.1051/vetres/2009011 19284965 PMC2695021

[B2] Eicher SC Dehio C Bartonella entry mechanisms into mammalian host cells Cell Microbiol 2012 14 1166 1173 10.1111/j.1462-5822.2012.01806.x 22519749

[B3] Van Audenhove A Verhoef G Peetermans WE Boogaerts M Vandenberghe P Autoimmune haemolytic anaemia triggered by Bartonella henselae infection: a case report Br J Haematol 2001 115 924 925 10.1046/j.1365-2141.2001.03165.x 11843827

[B4] Maggi RG Mascarelli PE Pultorak EL Hegarty BC Bradley JM Mozayeni BR Breitschwerdt EB Bartonella spp. bacteremia in high-risk immunocompetent patients Diagn Microbiol Infect Dis 2011 71 430 437 10.1016/j.diagmicrobio.2011.09.001 21996096

[B5] VanderHeyen TR Yong SL Breitschwerdt EB Maggi RG Mihalik AR Parada JP Fimmel CJ Granulomatous hepatitis due to Bartonella henselae infection in an immunocompetent patient BMC Infect Dis 2012 12 17 10.1186/1471-2334-12-17 22269175 PMC3287964

[B6] Correa FG Pontes CL Verzola RM Mateos JC Velho PE Schijman AG Selistre-de-Araujo HS Association of Bartonella spp. bacteremia with Chagas cardiomyopathy, endocarditis and arrhythmias in patients from South America Braz J Med Biol Res 2012 45 644 651 10.1590/S0100-879X2012007500082 22584639 PMC3854270

[B7] Yamashita H Ubano M Uesaka Y Kunimoto M A 34 year old woman with cat scratch disease who developed encephalopathy Rinsho Shinkeigaku 2012 50 576 580 22975857 10.5692/clinicalneurol.52.576

[B8] Chomel BB Boulouis HJ Maruyama S Breitschwerdt EB Bartonella spp. in pets and effect on human health Emerg Infect Dis 2006 12 389 394 10.3201/eid1203.050931 16704774 PMC3291446

[B9] Chomel BB Kasten RW Bartonellosis, an increasingly recognized zoonosis J Appl Microbiol 2010 109 743 750 10.1111/j.1365-2672.2010.04679.x 20148999

[B10] Billeter SA Levy MG Chomel BB Breitschwerdt EB Vector transmission of Bartonella species with emphasis on the potential for tick transmission Med Vet Entomol 2008 22 1 15 10.1111/j.1365-2915.2008.00713.x 18380649

[B11] Dehio C Infection associated type IV secretion system of Bartonella and their diverse roles in host cell interaction Cell Microbiol 2008 10 1591 1598 10.1111/j.1462-5822.2008.01171.x 18489724 PMC2610397

[B12] Breitschwerdt EB Suksawat J Chomel B Hegarty BC The immunologic response of dogs to Bartonella vinsonii subspecies berkhoffii antigens: as assessed by Western immunoblot analysis J Vet Diagn Invest 2003 15 349 354 10.1177/104063870301500408 12918816

[B13] Breitschwerdt EB Maggi RG Duncan AW Nicholson WL Hegarty BC Woods CW Bartonella species in blood of immunocompetent persons with animal and arthropod contact Emerg Infect Dis 2007 13 938 941 10.3201/eid1306.061337 17553243 PMC2792845

[B14] Breitschwerdt EB Maggi RG Lantos PM Woods CW Hegarty BC Bradley JM Bartonella vinsonii subsp. berkhoffii and Bartonella henselae bacteremia in a father and daughter with neurological disease Parasit Vectors 2010 3 29 37 10.1186/1756-3305-3-29 20377863 PMC2859367

[B15] Diniz PPVP Maggi RG Schwartz DS Cadenas MB Bradley JM Hegary B Breitschwerdt EB Canine bartonellosis: Serological and molecular prevalence in Brazil and evidence of co-infection with Bartonella henselae and Bartonella vinsonii subsp. berkhoffii Vet Res 2007 38 697 710 10.1051/vetres:2007023 17583666

[B16] Duncan AW Maggi RG Breitschwerdt EB A combined approach for the enhanced detection and isolation of Bartonella species in dog blood samples: Pre-enrichment culture followed by PCR and subculture onto agar plates J Micrbiol Meth 2007 69 273 281 10.1016/j.mimet.2007.01.010 17346836

[B17] Maggi RG Duncan AW Breitschwerdt EB Novel chemically modified liquid medium that will support the growth of seven bartonella species J Clin Microbiol 2005 43 2651 2655 10.1128/JCM.43.6.2651-2655.2005 15956379 PMC1151927

[B18] Breitschwerdt EB Maggi RG Mozayeni BR Hegarty BC Bradley JM Mascarelli PM Isolation or PCR amplification of Bartonella koehlerae from human blood Parasit Vectors 2010 3 76 10.1186/1756-3305-3-76 20735840 PMC2936392

[B19] Cadenas MB Maggi RG Diniz PP Breitschwerdt KT Sontakke S Breitschwerdt EB Identification of bacteria from clinical samples using Bartonella alpha-Proteobacteria growth medium J Microbiol Methods 2007 71 147 155 10.1016/j.mimet.2007.08.006 17889384

[B20] Jeyaprakash A Hoy MA Allsopp MH Bacterial diversity in worker adults of Apis mellifera capensis and Apis mellifera scutellata (Insecta: Hymenoptera) assessed using 16 rRNA sequences J Invertebr Pathol 2003 84 96 103 10.1016/j.jip.2003.08.007 14615218

[B21] Ullrich K Saha N Lake S Neuroretinitis following bull ant sting BMJ Case Reports 2012 10 1136 bcr-2012-006338, Published XXX 10.1136/bcr-2012-006338 PMC4543349 22865803

[B22] Maggi RG Mozayeni BR Pultorak EL Hegarty BC Bradley JM Correa M Breitschwerdt EB Bartonella spp. bacteremia and rheumatic symptoms in patients from Lyme Disease-endemic region Emerg Infect Dis 2012 18 783 791 22516098 10.3201/eid1805.111366 PMC3358077

[B23] Breitschwerdt EB Maggi RG Farmer P Mascarelli PE Molecular evidence of perinatal transmission of Bartonella vinsonii subsp. berkhoffii and B. henselae to a child J Clin Microbiol 2010 48 2289 2293 10.1128/JCM.00326-10 20392912 PMC2884525

[B24] Breitschwerdt EB Mascarelli PE Schweickert LA Maggi RG Hegarty BC Bradley JM Woods CW Hallucinations, sensory neuropathy, and peripheral visual deficits in a young woman infected with Bartonella koehlerae J Clin Microbiol 2011 49 3415 3417 10.1128/JCM.00833-11 21734026 PMC3165616

[B25] Lee TH Montalvo L Chrebtow V Busch MP Quantification of genomic DNA in plasma and serum samples: higher concentrations of genomic DNA found in serum than in plasma Transfusion 2001 41 276 282 10.1046/j.1537-2995.2001.41020276.x 11239235

[B26] Korkick DL Breitschwerdt EB Relapsing bacteremia after blood transmission of Bartonella henselae to cats Am J Vet Res 1997 58 492 497 9140557

[B27] Mavris M Saenz H Monteil M Boulouis HJ Dehio C Vayssier-Taussat M Characterization of genes involved in long-term bacteremia in mice by Bartonella birtlesii Ann NY Acad Sci 2005 1063 312 314 10.1196/annals.1355.050 16481533

[B28] Mascarelli PE Iredell JR Maggi RG Weinberg G Breitschwerdt EB *Bartonella* species bacteremia in two patients with epithelioid hemangioendothelioma J Clin Microbiol 2011 49 4006 4012 10.1128/JCM.05527-11 21918021 PMC3209129

[B29] Krause I Wu R Sherer Y Patanik M Peter JB Shoenfeld Y In vitro antiviral and antibacterial activity of commercial intravenous immunoglobulin preparations–a potential role for adjuvant intravenous immunoglobulin therapy in infectious diseases Transfus Med 2002 12 133 139 10.1046/j.1365-3148.2002.00360.x 11982967

[B30] Massei F Gori L Taddeucci G Macchia P Maggiore G Bartonella henselae infection associated with Guillain-Barre syndrome Pediatr Infect Dis J 2006 25 90 91 10.1097/01.inf.0000195642.28901.98 16395116

[B31] Talukder RK Sutradhar SR Rahman KM Uddin MJ Akhter H Guillian-Barre syndrome Mymensingh Med J 2011 20 748 756 22081202

[B32] Chomel BB Kasten RW Floyd-Hawkins K Chi B Yamamoto K Roberts-Wilson J Gurfield AN Abbott RC Pedersen NC Koehler JE Experimental transmission of *Bartonella henselae* by the cat flea J Clin Microbiol 1996 34 1952 1956 8818889 10.1128/jcm.34.8.1952-1956.1996 PMC229161

[B33] Telford SR 3rd Wormser GP Bartonella spp. transmission by ticks not established Emerg Infect Dis 2010 16 379 384 10.3201/eid1603.090443 20202410 PMC3322007

[B34] Cotte V Bonnet S Le Rhun D Le Naour E Chauvin A Boulouis HJ Lecuelle B Lilin T Vayssier-Taussat M Transmission of Bartonella henselae by Ixodes ricinus Emerg Infect Dis 2008 14 1074 1080 10.3201/eid1407.071110 18598628 PMC2600320

[B35] Reis C Cote M Le Rhun D Lecuelle B Levin ML Vayssier-Taussat M Bonnet SI Vector competence of the tick *Ixodes ricinus* for transmission of *Bartonella birtlesii* PLoS Negl Trop Dis 2011 5 e1186 10.1371/journal.pntd.0001186 21655306 PMC3104967

[B36] Dietrich F Schmidgen T Maggi RG Richter D Matuschka FR Vonthein R Breitschwerdt EB Kempf VA Prevalence of Bartonella henselae and Borrelia burgdorferi sensu lato DNA in Ixodes ricinus ticks in Europe Appl Environ Microbiol 2010 76 1395 1398 10.1128/AEM.02788-09 20061459 PMC2832386

[B37] Tsai YL Lin CC Chomel BB Chuang ST Tsai KH Wu WJ Huang CC Yu JC Sung MH Kass P Chang CC Bartonella infection in shelter cats and dogs and their ectoparasites Vector Borne Zoonotic Dis 2011 11 1023 1030 10.1089/vbz.2010.0085 21142966

[B38] Holden K Boothby JT Kasten RW Chomel BB Co-detection of Bartonella henselae, Borrelia burgdorferi, and Anaplasma phagocytophilum in Ixodes pacificus ticks from California, USA Vector Borne Zoonotic Dis 2006 6 99 102 10.1089/vbz.2006.6.99 16584332

